# Regional distribution of mercury in sediments of the main rivers of French Guiana (Amazonian basin)

**DOI:** 10.1186/2193-1801-3-322

**Published:** 2014-06-26

**Authors:** Valérie Laperche, Jennifer Hellal, Régine Maury-Brachet, Bernard Joseph, Pierre Laporte, Dominique Breeze, François Blanchard

**Affiliations:** BRGM, D3E, Water, Environment and Ecotechnology Division, 3 avenue Claude Guillemin, BP 36009, 45060 Orléans, France; UMR CNRS 5805 EPOC – OASU Station Marine d’Arcachon, Université Bordeaux 1, Place du Docteur Bertrand Peyneau, 33120 Archachon, France; French Geological Survey (BRGM), Direction régionale Guyane, Domaine de Suzini, Route de Montabo, BP 552, 97333 Cayenne cedex, France

**Keywords:** Mercury, French Guiana, Sediments, Gold-mining

## Abstract

Use of mercury (Hg) for gold-mining in French Guiana (up until 2006) as well as the presence of naturally high background levels in soils, has led to locally high concentrations in soils and sediments. The present study maps the levels of Hg concentrations in river sediments from five main rivers of French Guiana (Approuague River, Comté River, Mana River, Maroni River and Oyapock River) and their tributaries, covering more than 5 450 km of river with 1 211 sampling points. The maximum geological background Hg concentration, estimated from 241 non-gold-mined streams across French Guiana was 150 ng g^-1^. Significant differences were measured between the five main rivers as well as between all gold-mining and pristine areas, giving representative data of the Hg increase due to past gold-mining activities.

These results give a unique large scale vision of Hg contamination in river sediments of French Guiana and provide fundamental data on Hg distribution in pristine and gold-mined areas.

## Introduction

A clear link has been established between mercury (Hg) contamination in the Amazon region and gold mining activities due to the use of metallic Hg in the mining process (Veiga et al. 1999), however, in some cases, the link between Hg contamination (soil, sediment, fish or human) and gold mining is not always clear. For example, Quenel et al. (2007) found high levels of Hg in the hair of the Amerindian population of Trois Sauts in the upper Oyapock River (French Guiana); yet there is no gold mining in this area. Prior to Quenel’s study, Hg contamination has also been reported in riparian populations in remote areas of the upper Negro River (Silva-Forsberg et al. 1999), the Tapajos River (Castilhos et al. 1998), the Apiacas Reserve (Barbosa et al. 1997) and the Amapas (Bidone et al. 1997).

Hg concentrations in Amazonian soils are often high, even in areas far from anthropic sources (Roulet et al. 1998; Lechler et al. 2000; Fadini and Jardim 2001, De Oliveira et al. 2001).

Lithogenic Hg accumulates in soil due to the release of more mobile elements during the weathering of source rocks (iron oxides) (Grimaldi et al. 2001 Roulet et al. 1998). The age of tropical soils (several million years; very high compared to the ages of soils in temperate areas) amplifies this phenomenon. Atmospheric Hg results from degassing of the Earth’s crust via the oceans and volcanoes, and is deposited on the top soil either directly or after canopy leaching (Mason et al. 1994). Lithogenic and atmospheric Hg stored in soil are primarily present in a divalent form (Hg(II)) (Schuster 1991).

One of the main sources of anthropic Hg is metallic Hg which is used to amalgamate gold in gold mining processes. In 2005, in French Guiana, the gold production came for a quarter from primary gold ores (for 4 extraction sites only) and three quarters from alluvial placer deposits. At this time all mining techniques used Hg in the process (since 01/01/2006 the use of Hg is banned in France). Picot et al. (1993) estimated a Hg/Au ratio of 1.37. Using this ratio and the quantity of gold declared in 2005, the total losses of Hg can be estimated to be around 286 T in French Guiana.

Hg is present in river waters in very low concentrations, much lower than the French drinking water standards (1 μg L^-1^) or the Maximum Contaminant Level (MCL) of 2 μg L^-1^ (EPA), and presents no risk for direct consumption or for swimming. Moreover, 99% of the Hg present in Guiana river water is in the inorganic form (Boudou et al. 2006; Sampaio Da Silva et al. 2009; Roulet et al. 1999). However, inorganic Hg may be converted to organic forms (methylated forms such as monomethylmercury: MMHg), which are highly toxic and potential neurotoxins (World Health Organisation WHO 1990). Conversion to MMHg is generally a microbial process, through the action of sulfate or iron reducing bacteria (Compeau and Bartha 1985; King et al. 2002; Fleming et al. 2006). Hg biomagnification along the food webs, principally based on cumulative transfers of the methylated form, can lead to extremely high Hg concentrations in piscivorous species at the top of the trophic networks (Boudou and Ribeyre 1997; Morel et al. 1998). Soil erosion also contributes to contaminating the food chain by transporting high quantities of Hg(II) associated with particles towards bottomlands and rivers, where biogeochemical conditions favor Hg methylation (Morel et al. 1998; Ullrich et al. 2001).

In 1994, the French National Public Health Network (InVS) reported significant Hg exposure of native Amerindians in French Guiana through fish consumption (Cordier et al. 1997). In response, InVS, in collaboration with the National Scientific Research Center (CNRS) and the health authorities, launched several studies which main objectives were to (i) quantify Hg intake with food and (ii) identify the principal fish species with the highest Hg levels. The overall river contamination, notably sediments, was not considered in these studies.

In 2004, a monitoring program (Regional distribution of mercury in sediments and fishes of six rivers of French Guiana) was established in order to assess Hg contamination of the human populations of French Guiana. This program aimed to generate a database of Hg concentrations in sediments and fish covering as much of the territory of French Guiana as possible. The project focused on total Hg concentrations. Indeed, 97 to 100% of Hg in fish is under the MMHg form and thus is equivalent to total Hg contents (Maury-Brachet et al. 2006). Sediment sampling and analyses (n = 1 211) were carried out by the BRGM (this study) on five of the six Rivers, while the CNRS focused on Hg concentrations in fish (n = 974) in all six rivers. The originality of this study lies in its large scale regional approach whereas most of the previous studies are focused on one site or river. Moreover, the use of the same sampling strategy and analytical method on all samples enables data comparison over the whole study area. This study presents results of total Hg in sediments and compares them with values measured in other Amazonian regions to distinguish the anthropogenic impact from the natural background level. The final goal was to establish, on the scale of French Guiana, a Hg contamination map, usable by the Guyanese community to take into account environmental impacts in the development of French Guiana.

## Methods

### Study area

**Figure 1:**
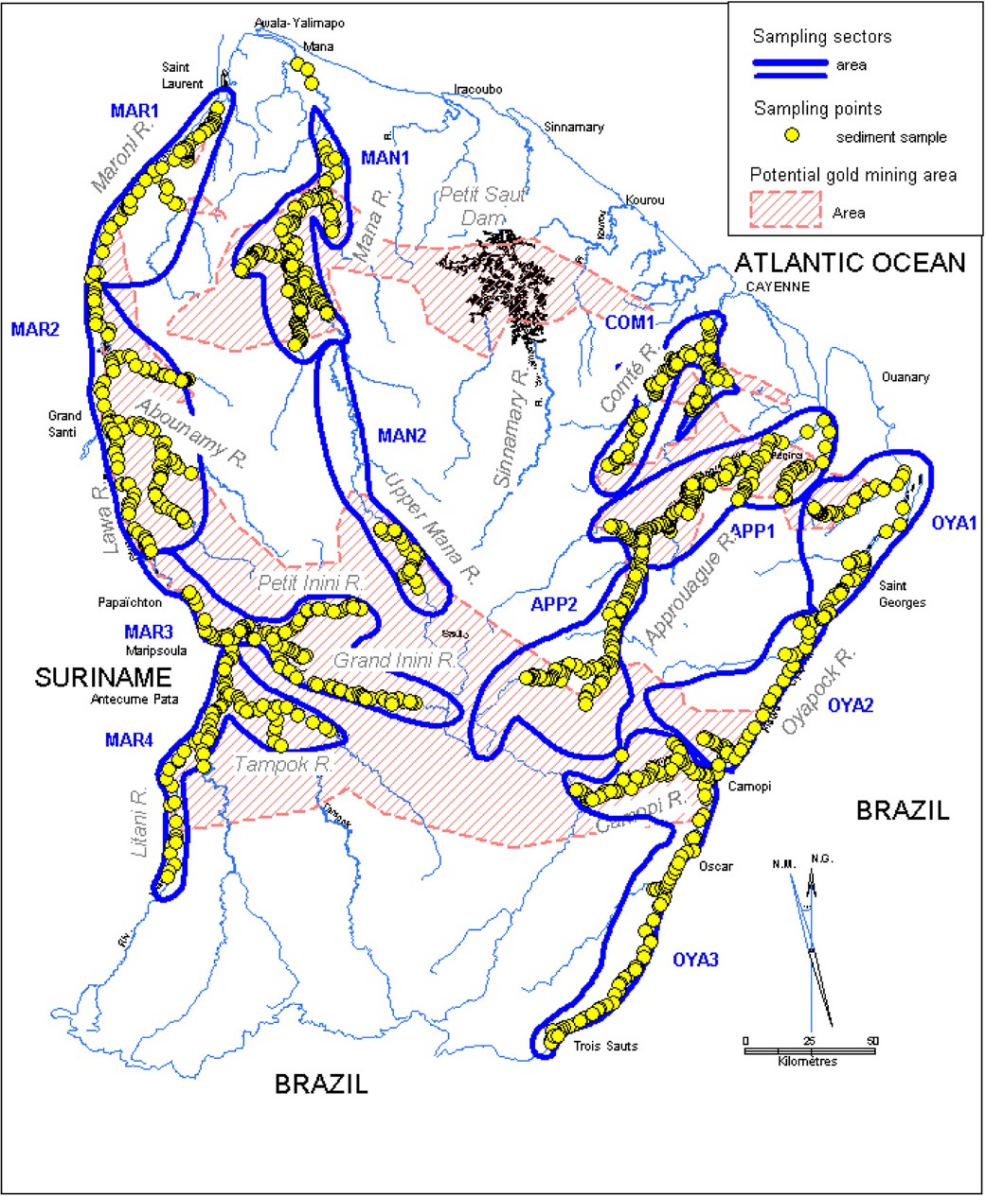
**French Guiana map with sampling points**, **sampling sectors and the potential gold mining areas.**

French Guiana is situated in northern South America within 2-6°N and 51°30′-54°30′W with Surinam to the west, Brazil to the east and south and the Atlantic Ocean to the north (Figure 1). The total area of 87 500 km^2^ (Collectif 2002) is approximately 96% tropical rainforest. Most of the gold deposits are epigenetic, sited in the proximity of major geological structures, and linked to low and medium metamorphic grade granitoid-greenstone belts (Milesi et al. 1995; Voicu et al. 2001). Weng et al. (2006) established a map of the potential maximum surface touched by the gold bearing extraction by taking into account parameters such as the geological data, the map of the known gold mining sites (BRGM Inventory of the mining resources of the French territory, 1970–1995) and the ancient and recent placer mining (legal and illegal). These areas cover a total surface of almost 29 000 km^2^ (Figure 1), there are two large bands parallel to the coast, one from Saint George to Saint Laurent and the second from Camopi to Grand Santi.

### Sampling area and method

The geographical sectors studied cover most of the hydrographical axes of French Guiana navigable by a motorboat, including their tributaries, whether they are gold mined or not, in order to be able to distinguish the natural geochemical background from the anthropogenic activities. This study systematically sampled all the representative lithologies of French Guiana to compare the Hg levels in mined areas to pristine areas. From September 2005 to November 2006, 1 211 samples were collected along approximately 5 450 km of five main rivers (Approuague River, Comté River, Mana River, Maroni River and Oyapock River) and theirs tributaries. Sampling locations were semi-uniformly spaced at approximately 1, 5 or 10 km intervals. The spacing between sampling sites was defined to allow the collection of a maximum number of samples in the available time. The 1 km step was used for the zones of increased interest (vicinity of villages or communes, gold mining sites and at the mouths of tributary’s draining basins characterized by extensive mining operations); the 5 km step was adopted in all other sectors. Exceptionally, a step of 10 km was used in the estuaries of the large rivers (zones generally subjected to the tide). The location of each of the sampling sites was recorded in the field with a GPS.

The different sampling steps aimed to evaluate i) the potential Hg sources: sampling downstream from the known gold mining sites, if possible with the up and downstream of these sites (1 km steps), and ii) Hg dispersion along the major drains: regular 5 km sampling step on the large rivers.

The watershed of the five rivers was cut into 12 sampling areas: Approuague River (APP 1 et 2), Comté River (COM 1), Mana River (MAN 1 et 2), Maroni River (MAR 1 à 4), Oyapock River (OYA 1 à 3) (Figure 1). The sampling areas were defined on the basis of logistic criteria (duration of the mission, oil autonomy, starting place and withdrawal), socio-politic criteria, and quality of the services (participation of the local communities).

At each sampling site, samples were collected from just above the low water line during low tide on river banks where finer sediment tends to accumulate. As these sediments were recently deposited during high tides, it is assumed that they provide geochemical information that reflects the last season’s conditions. To eliminate the pieces of leaf and twigs, all the sediments collected with a stainless steel grab were passed through a 500 μm sieve directly after sampling. None of the 1 211 sediment samples collected contained coarse sand (500–2000 μm). Samples were stored in polyethylene bottles and shipped to the laboratory in Orléans (France) within one or two weeks depending on the sampled area.

In order to sample all situations, different types of rivers were sampled: main rivers, their tributaries, medium rivers and streams (or creeks). Out of the 1 211 samples, 401 samples were collected on the banks of the main rivers (11 rivers are considered as main rivers: the large rivers such as Maroni, Mana, Comté, Approuague and Oyapock but also some of the large tributaries of these rivers such as Abounamy, Camopi, Tampok or Grand Inini). 327 samples were collected on the banks of the medium rivers (tributaries of the main rivers, 22 of which 8 have been gold mined); 241 were collected on the banks of gold-mined-streams and 242 on the banks of un-mined-streams.

Among the 1 211 samples, 387 (1/3 of the total samples) were taken in rivers which waters were very turbid and 33 samples were taken in intertidal areas still under the influence of the Amazon River (Huynh et al. 1996).

### Analytical techniques

All samples were homogenized and sub-samples were dried at 40°C in an oven then ground (<80 μm) in an agate mortar before analysis. Total Hg was determined by AAS (Atomic Absorption Spectrometry) using a RA 915+ equipped with the pyrolysis attachment (Lumex Society, Russia). All Hg results are given in ng of Hg per g of dry sediment (ng g^-1^).

Moisture was determined by weight loss in separate sub-samples by drying at 105°C overnight.

During the second mission on the Approuague River (APP2), water turbidity was measured with a field turbidimeter (TN100, Eutech) at each of the 80 sampling points. Turbidity is reported as Nephelometric Turbidity Units (NTU).

### Quality control of Hg determinations

The drift in Hg determinations was followed every ten samples by analyzing standard reference material (Standard NCS DC78301 at 0.22 ± 0.04 μg g^-1^ Hg from the National Analysis Center, China and standard LGC6156 at 10.1 ± 1.6 μg g^-1^ Hg from the National Measurement Institute for chemical and bio analytical measurements, UK) to evaluate the analytical accuracy within 5%. The reproducibility of the method was 3 to 5% except for concentrations between 250 and 1 500 ng kg^-1^ where the reproducibility was 1.2 to 2.3%.

About 10% of the samples were also analyzed by CV-AFS in an independent laboratory (Chemex ALS, Canada) to control Lumex results.

### Statistical analysis

Differences between Hg concentrations were determined with a nonparametric kruskal-Wallis test using the R software and *pgirmess* package (R Development Core Team, 2004; Siegel and Castellan 1988).

## Results and discussion

### Textural classes of the sediment

Based on the revised textural classification scheme of gravel-free muddy sediment proposed by Flemming (2000) using the sand/mud ratios and the textural definition of mud as all sediments finer than 62.5 μm (silt + clay), the 1 211 sediments collected can be ranged in 5 classes (Table 1). Most of the sediments (87%) have a texture between muddy sand and slightly sandy mud; not a single sample has a sandy texture.

**Table 1:** **Percentage of samples for the 6 textural classed based on mud content as defined by Flemming** (2000)

Mud content (%)	Textural class	Samples (%)
< 5	Sand	0.0
5-25	Slightly muddy sand	7.1
25-50	Muddy sand	29.3
50-75	Sandy mud	31.3
75-95	Slightly sandy mud	26.3
> 95	Mud	6.0

### Background Hg concentrations

Several studies (Roulet et al. 1998, Artaxo et al. 2000, Carmouze et al. 2002, Muresan 2006) have shown that Hg levels in Amazonian soils are high compared to temperate zones, mainly due to soil pedogenesis and high atmospheric depositions. Thus the soils constitute an important reservoir of naturally accumulated Hg that can be mobilized through naturel or anthropic erosion (deforestation, gold-mining) and induce increased export of terrestrial Hg to aquatic ecosystems.

Before looking at the regional distribution of Hg in river sediments of French Guiana, it is first important to evaluate the natural background levels of Hg (“natural background” is widely used to infer “background” levels reflecting natural processes uninfluenced by human activities; it is more realistic to view background as a range rather than an absolute value). The background level was evaluated from 51 samples collected on the Upper Oyapock (south of the Oscar’s village, Figure 1). This part of the river and its tributaries are known to be free of alluvial gold mining activity. The mean Hg concentration in the muddy sediment of the Upper Oyapock is 100 ± 30 ng g^-1^. This value is close to the one calculated from the 241 sediments collected in the un-gold-mined-streams (NGWS in Table 2) of the whole of French Guiana (108 ± 42 ng g^-1^). It was not possible to estimate a background level for each NGMS because of the small number of data on some streams (n = 3); a background level was therefore calculated per sector. The background levels vary between 70 ± 33 ng g^-1^ and 122 ± 32 ng g^-1^ depending on geology and locality of the sector.

**Table 2:** **Mercury concentration statistics (ng g** ^**-1**^ **) calculated separately for the total data set, each main river, the No Gold Mined Streams (NGMS) and the Gold Mined Streams (GMS)**

Hg (ng.g ^-1^)	French Guiana	Comté River	Approuague River	Mana River	Maroni River	Oyapock River	NGMS	GMS
Minimum	12	31	12	24	18	22	19	30
Maximum	1 320*	237	1 320*	768	465	775	231	930
Median	117	91	129	122	129	100	110	154
Medium	152	98	238	143	139	114	108	190
n^1^	1 211	105	255	212	385	254	241	242
σ	438	45	85	100	69	937	42	137

1: number of samples, *maximum after removal of two aberrant values from the Approuague River (10.05 and 11.2 μg g^-1^).

These background values are on the same level of magnitude to the background levels measured by Charlet et al. (2003) on the Litani River (109 ± 30 ng g^-1^) situated in the MAR4 sector and Roulet and Lucotte (1995) in French Guiana (180–320 ng g^-1^). They are also comparable to other observations in the Amazonian basin such as the Madeira watershed (41–439 ng g^-1^; Lechler et al. 2000), the Tapajos watershed (90–210 ng g^-1^; Roulet et al. 1998) or the Rio Negro watershed (81–320 ng g^-1^; Fadini and Jardim, 2001). Thus, the maximum value for the background level of Hg in sediment was estimated to be 150 ng g^-1^, except in the estuary areas where sediments have a low Hg level (around 50 ng g^-1^) due to the influence of Amazon deposits.

### Gold-mined versus non-gold-mined streams

**Figure 2:**
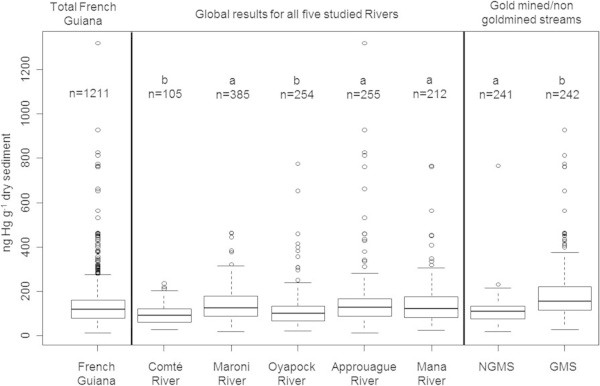
**Boxplot of mercury sediment contents for the whole of French Guiana, the five main rivers studied and gold** **-** **mined and non** **-** **gold** **-** **mined streams (GMS and NGMS).** Two aberrant points measured along the Approuague River were removed from the analysis (values of 10.05 and 11.2 μg g^-1^, respectively). Statistical differences between groups according to a Kruskal-Wallis test are indicated by the letters above the boxplots as well as the number or samples (n).

The average level in the NGMS (108 ± 42 ng g^-1^), i.e. the background level, is significantly lower (Kruskal-Wallis, p-value < 0.001) and less variable than the Hg concentrations in sediments of the Gold Mined Streams (GMS), (190 ± 137 ng g^-1^) (Table 2 and Figure 2).

**Figure 3:**
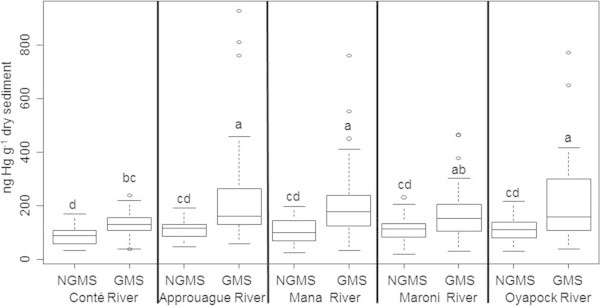
**Boxplot comparing Hg contents in sediments from the gold**-**mined and non**-**gold**-**mined streams (GMS and NGMS) from the five main river sectors.** Two aberrant points measured along the Approuague River were removed from the analysis (values of 10.05 and 11.2 μg g^-1^, respectively). Statistical differences between groups according to a Kruskal-Wallis test are indicated by the letters above the boxplots as well as the number or samples (n).

Kruskal-Wallis tests were also run between the GMS and NGMS for each sub-sector and showed significant differences. Thus, data was grouped for each river sector and tests were repeated on this data (Figure 3). These results showed that whatever the region considered, there was always significantly higher Hg concentrations in the sediments from gold-mined streams compared to non-gold-mined streams.

### Regional distribution of Hg concentrations in sediments

**Figure 4:**
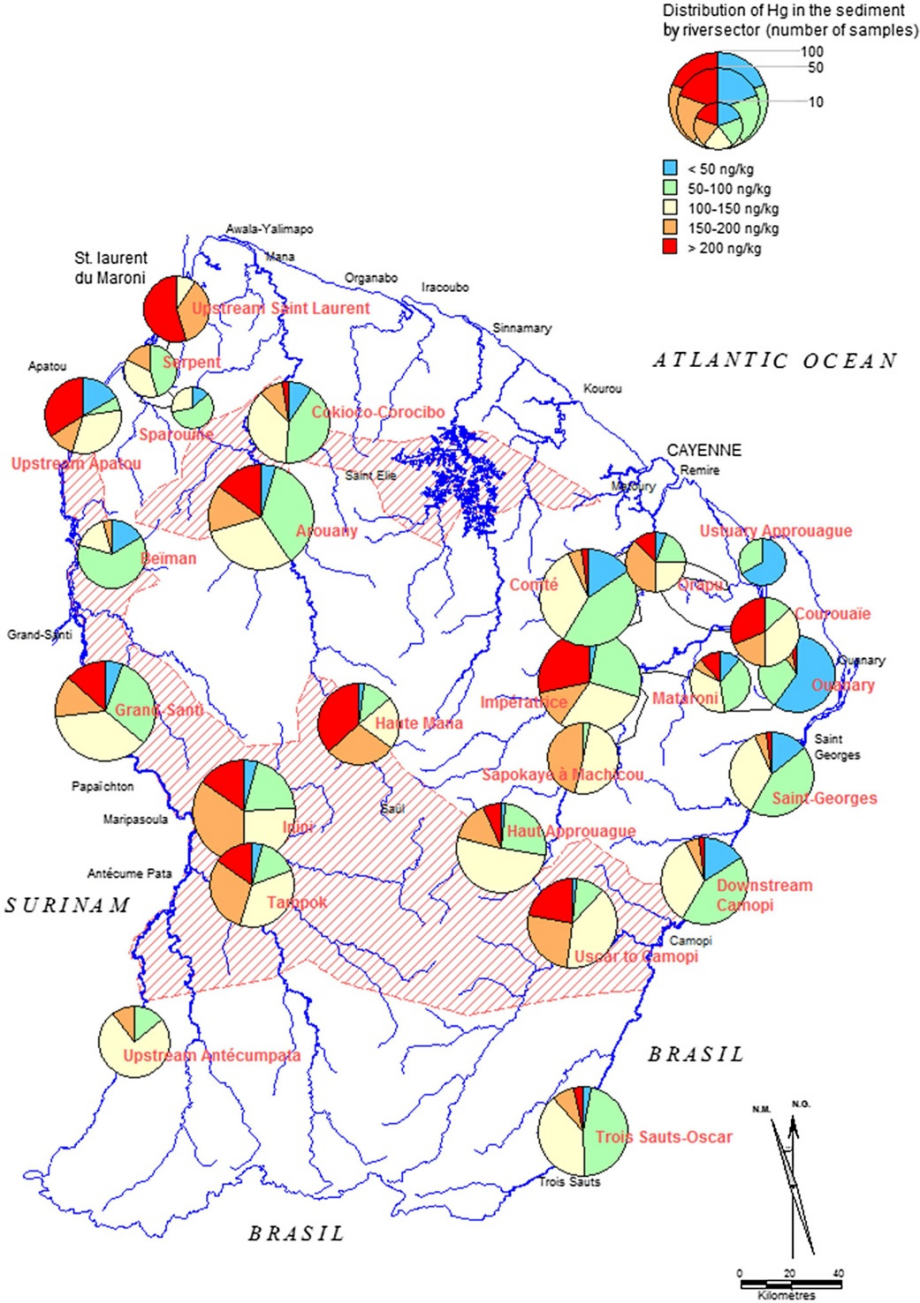
**Distribution of Hg in sediments by river sector.**

Hg concentrations in the samples ranged from 12 to 11 200 ng g^-1^ (Table 2). 70% of the sediments have Hg concentrations lower than the maximal value of the background level: 150 ng g^-1^ (Figure 4). The 6 highest values were measured in sediments of the Approuague River (810, 830, 930, 1320, 10 050 and 11 200 ng g^-1^ from “Impératrice”, “Mataroni” and “Haut-Approuague” sectors). The minimal values, between 10 and 30 ng g^-1^ were measured for the Comté and Oyapock rivers in mud samples close to the estuary. For the other rivers, the sediments with low Hg levels were taken either in or just downstream from granitic areas. Significant differences (Kruskal-Wallis test p-value < 0.001) were shown between Hg concentrations in the Comté and Oyapock rivers and the other three main rivers (Mana, Maroni & Approuague) which concentrations were globally higher by up to 40% (Figure 2, Table 2).

The whole dataset is presented on the map shown in Figure 4, where the results are presented by river sectors and not point by point. Each river sector covers one or a few fishing spots (max. 3), (Laperche et al., 2007) of a same catchment area.

*The Oyapock River* has been divided into five river sectors. (i) From Trois Sauts to the Yaloupi stream (south of Oscar’s village: “Trois Sauts-Oscar” on Figure 4), the sector is free of gold mining activity and water of the Oyapock River was clear to clouded. Water was always clear in the tributaries of the Oyapock River (Moulou-moulou, Anapokéa and Yaloupi streams). Hg concentration in these sediments is always lower than 130 ng g^-1^ (Figure 4). (ii) From Yaloupi stream to Maripa village (“Oscar to Camopi”), the sector is, or was, gold mined towards the end of the 19^th^ century. At the time of sampling Camopi River was gold mined and several high values were measured on the mining sites (650 and 775 ng g^-1^). All the high values were found in sediments collected in turbid waters of GMS (Alicorne, Tampack and Alikéné). Thus it is possible to distinguish two types of streams:

-  Streams with turbid to highly turbid water, downstream or close to the mines. Sediments are yellow to ochre and their Hg concentrations are between 200 and 800 ng g^-1^.
-  Streams with clear to clouded water. Sediments are brown to grey and their Hg concentrations are lower than 150 ng g^-1^.

(iii) From Camopi to Maripa’village (south of Saint Georges: “Downstream Camopi”), the mean value is 94 ng g^-1^ but a few samples collected in the Oyapock River have higher Hg concentrations (up to 208 ng g^-1^). This sector is not gold mined anymore but up to 50 dredges were in activity until the eighties. (iv) From Maripa to the estuary (“Saint Georges”), this sector is not gold mined on the French side but it is slightly on the Brazilian side. The mean value is 97 ng g^-1^. They are lower in the north of Nouvelle Alliance (<40 ng g^-1^) but this zone corresponds to the limit of mud deposition from the Amazon River. (v) The Ouanary River is gold mined but ¾ of this river is under the influence of the tide; the major part of the sediments is mixed or covered with marine mud thus giving low Hg concentrations (50 ± 22 ng g^-1^).

*The Approuague River* has been divided into six river sectors. (i) The Upper Approuague (“Haut Approuague”) was much more gold mined in the past than nowadays. In this zone, the mean value is 142 ng g^-1^. Four samples have higher concentrations (up to 1 320 ng g^-1^ close to Sapokaï) that can be explained by the presence of dredges in the past. (ii) The zone between Sapokaï and the Machicou stream (“Sapokaye to Machicou”) is granitic and the sediments have a Hg concentration close-to or lower-than the background level. (iii to v) The lower Approuague sector (Impératrice, Mataroni and Courouaïe: “Impératrice”) has been heavily gold mined since the end of the 19^th^ century, with numerous dredges. Indeed it is of the same geological formation as the Belizon mountain which is presently a large gold mining reserve. High Hg concentrations were found along the river; the 43 samples with a Hg concentration higher than 200 μg g^-1^ represent 33% of the samples from this sector, whereas 14% of the samples from the whole territory have Hg concentrations higher than 200 μg g^-1^. The two highest Hg concentrations (>10 000 ng g^-1^) were measured in sediments collected in the richest gold-nugget area of French Guiana (Impératrice); (vi) From Régina to Guisan bourg (“Approuague Estuary”), the area is not gold mined. The highest Hg concentration is 51 μg g^-1^ and it is lowest to the north (<40 ng g^-1^) where the river is under the influence of the tide and the Amazon River sediments.

*The Comté sectors* are composed by the Comté and the Orapu rivers. Hg concentrations are lower than the background level. The mean Hg concentration of sediments collected in the NGMS is much lower than the Hg concentration of sediments collected in the GMS (82 ± 39 ng g^-1^ and 134 ± 55 ng g^-1^, respectively).

*The Mana River* can be divided into three river sectors separated by a granitic zone (Delor et al. 2001) not favorable for mud sampling (mostly pure quartz). (i) The Upper Mana (“Haute Mana”) is a heavily gold mined area. The lowest Hg concentration is 98 ng g^-1^ and 75% of the samples collected in the Upper Mana have a Hg concentration higher than 177 ng g^-1^. (ii) The river sector of the lower Mana is also an area with much gold mining, in particular, the Arouani River (“Arouany”) and (iii) the Mana River from the Arouani River to Cokioco and Corocibo rivers (“Cokioco-Corocibo”). The latter two rivers are slightly gold mined and the mean Hg concentrations in the sediments are 95 ± 52 ng g^-1^ and 96 ± 29 ng g^-1^, respectively. Upstream from the Arouani River, Hg concentrations are low (<50 ng g^-1^) but they increased rapidly downstream towards the placer “Délices” (~500 ng g^-1^), one of the biggest placer of French Guiana.

Hg concentrations from the junction of the Mana River with the Cokioco River to the estuary remain high (110 ± 36 ng g^-1^) compared to the estuaries of the other rivers. The Mana estuary is parallel (oriented NW-SE) to the coast, not perpendicular as for the other main rivers, and thus less favorable to marine mud deposition which dilutes continental Hg-contaminated deposits.

*The Maroni River* (border between Suriname and French Guyana) is composed by 3 rivers: the Litany, the Lawa and the Maroni). From upstream to the estuary, many gold-mined streams flow into the main rivers and many dredges are present on the rivers themselves. Therefore, the mean Hg concentrations increased from upstream to the estuary: from 128 ± 30 ng g^-1^ (Litani River), to 169 ± 63 ng g^-1^ (Lawa River) and 193 ± 53 ng g^-1^ (Maroni River). (i) The river sector from “upstream to Antécum Pata” is not gold mined on the French side but it is gold mined on the Surinamese side (Oulemali River). Hg concentrations are lower than the Background. (ii) The river sectors of Maripasoula (“Tampok” et “Inini”) with the Ouaqui, Grand Inini and Petit Inini rivers are gold mined (235 ± 70 ng g^-1^).

(iii)The “Grand Santi” area is composed of the main rivers (Maroni and Lawa) and their tributaries Grand Abounamy and Petit Abounamy as well as their streams. A few dredges are present on the main river and 62% of the sediments have an Hg concentration higher than the Background level. (v) From the North of the Beïman River to the estuary (“Upstream Saint Laurent”), there are many dredges on the Maroni River (especially in the Apatou area) and 90% of the samples have a Hg concentration higher than the Background level. On the opposite, 7% of the sediments from former gold mined tributaries have a Hg concentration higher than the Background level (“Beïman”, “Serpent” and “Sparouine” river sectors).

### Link between Hg concentrations and turbidity

**Figure 5:**
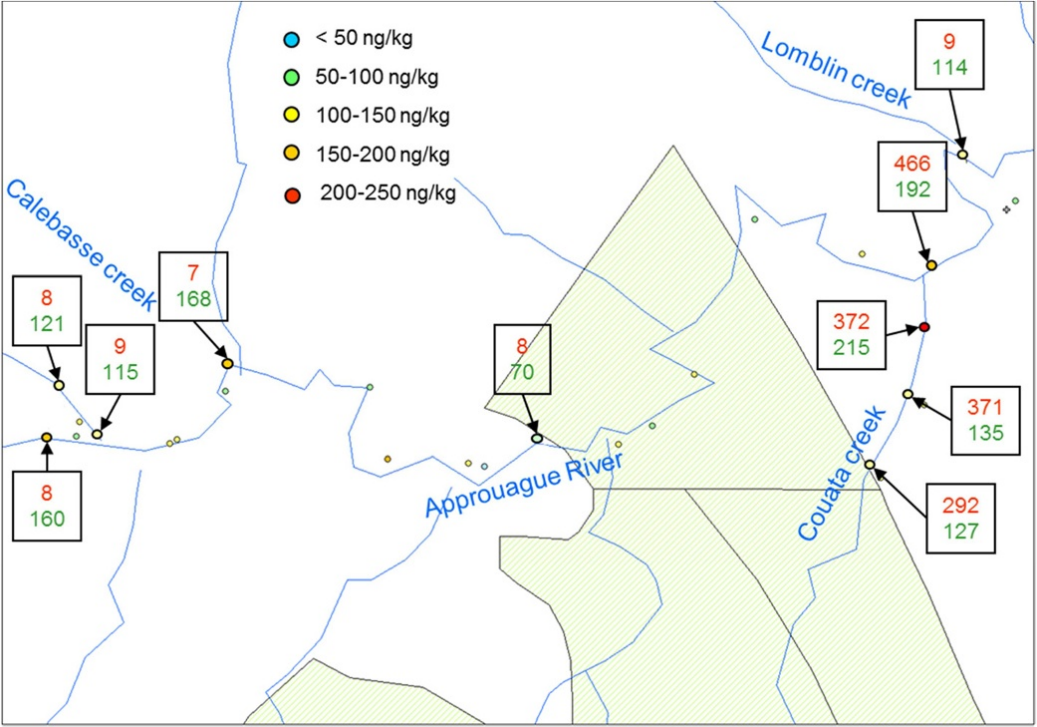
**Comparaison of Hg**-**sediment concentration and water turbidity along the Approuague River (in red**: **turbidity data in NTU and in green**: **Hg concentration in ng**/**kg).**

Most of the time, very turbid waters with ochre yellow colors are characteristic of upstream mining; it is therefore interesting to check if turbidity is actually correlated to Hg concentrations in sediments. This was carried out in 80 sampling spots. For example, during the second mission on the Approuague River, water turbidity was measured at each sampling point (Figure 5). On Calebasse creek, Hg concentrations in sediments are between 115 and 170 ng g^-1^ and the turbidity measurements are around 8 NTU. On Couata creek, turbidity measurements are much higher (290 to 470 NTU) but Hg sediment concentrations didn’t increase proportionally (130 to 215 ng g^-1^). On Lomblin creek, turbidity measurements are around 50 NTU, higher than in the first stream, but Hg concentration in sediments is lower (~70 ng g^-1^). The total turbidy database gave similar results (data not shown) to those on the Approuague. Thus it was not possible to establish a direct relationship between high Hg concentrations in sediments and sediment typology or water turbidity. Therefore, turbidity measurements are not a useful indicator of Hg contamination but are still a good indicator of the presence of active mines (Vigouroux et al. 2005).

For 7 km downstream from the Dorlin placer, one of the biggest placer of French Guiana, Hg concentrations in the sediment remained higher than 200 ng g^-1^. For the next 25 km until the Grand Inini River, they are between 200 and 150 ng g^-1^, and then along the following 25 km after the confluence of the two rivers, they are between 150 and 100 ng g^-1^. A similar decrease was observed downstream from « Dégrad Saint Léon », in the “Haute Mana” river sector on the Mana River. Here Hg concentrations start at ~200 ng g^-1^, then decrease to 100 ng g^-1^ along the 5–6 km of the Mana River. In this case, all the streams flowing down the Mana River are not gold mined, but beyond the “Deux Branches” stream, Hg concentrations of the sediments increased to 250 ng g^-1^. Along the 10 km, the streams (Marc and Marie streams) flowing down the Mana River are gold mined. Thus, it is possible to follow a progressive decrease of Hg concentrations in sediment along the 5–7 km of the river. The impacted river length depends, amongst other things, on the size and activity of the mining placer upstream but also on the activity on the streams flowing down the main river. These values can be compared with those measured on the exploitation site of “Saint Elie” (Grimaldi et al. 2001). Hg concentrations in sediments upstream from the placer, in the vicinity of the site, and downstream are 127, 2 489 and 549 ng g^-1^, respectively. There is an enrichment of Hg in the sediments downstream from the placers due to the transport of the finest particles.

All samples taken in rivers 20 or 30 km down from the placer do not show high Hg concentrations even if the waters are turbid as for the Sikini and the Lézard streams (97 ± 22 ng g^-1^ and 69 ± 15 ng g^-1^, respectively).

## Conclusion

The background Hg level in river sediments of the whole of French Guiana was estimated to be 108 ± 42 ng g^-1^, depending on the mud/sand ratio. Around 70% of sediments have Hg concentrations lower than 150 ng g^-1^. An important point is the good correlation between the areas presenting high Hg concentrations (>150 ng g^-1^) and the areas with strong gold bearing potential. In upstream areas such as « Trois Sauts » and « upstream Antecum Pata », 89% and 100% of Hg concentrations in sediments are lower than 150 ng g^-1^ whereas values < 150 ng g^-1^ represent only 65, 50, 48, 45 and 41% of samples in the gold mined areas « Haute Mana », « Inini », « Camopi », « Tampok » and « Impératrice », respectively (Table 2).

The extensive data set collected in the present study enables typical configurations to be identified:

-  gold mined areas (historical or present) with anomalous Hg concentrations (4–500 ng g^-1^ to 10 000 ng g^-1^);
-  rivers and streams with ochre yellow sediments close to mined sites with high Hg concentrations (>200 ng g^-1^);
-  rivers and streams with brown to grey sediment with low Hg concentrations (<150 ng g^-1^);
-  rivers close to the estuaries (marine sediment deposit areas) with very low Hg concentrations (<60 ng g^-1^);
-  granitic areas (sandy sediment) with very low Hg concentrations (10 to 50 ng g^-1^).

The increase of Hg in sediment downstream from mining areas is clearly related to gold-mining activity. Besides Hg contamination, impacts of gold mining are numerous: deforestation, changes in river regime and ecology due to sedimentation and flow modification, changes in landform and visual intrusion, land degradation due to inadequate rehabilitation after closure, land instability… Roulet et al. (1998) and Grimaldi et al. (2008) have shown that Amazonian soils, including areas far from anthropic sources, are often highly concentrated in Hg, therefore constituting an important reservoir of naturally accumulated Hg. In comparison, the inputs from gold mining could represent less than 3% of the Hg present in the first 20 cm of the soil (Roulet et al. 1999). These studies suggest that throughout the river valley, erosion of fine particles from the soil cover is responsible for the increased export of terrestrial Hg to aquatic ecosystems. Soil erosion can be caused by disturbance of a land surface: building and road construction, slash and burn practices, mining… There is no data on the inputs from gold mining in deeper soil horizons of the historical placers. From the ratio Hg/Au of 1.37, Picot et al. (1993), estimated the total Hg losses at 286 T in French Guiana (60% Hg_liq_ and 40% Hg_vap_) from 1857 to 2005. It is possible to have punctually very high Hg levels in soil on the historical placers. Since 01/2006, it is forbidden to use Hg in French Guiana, but reworking these historical placers could liberate large amounts of Hg. Gold mining dramatically increases soil erosion and the low settling rate of particles combined with their high Hg concentration implies a risk of Hg transfer to the hydrological network and the food chain (Boudou et al. 2006; Durrieu et al. 2005; Dominique 2006; Guédron 2008).
